# An expanded biomarker panel for the detection of prostate cancer from urine DNA

**DOI:** 10.1186/s40164-019-0137-x

**Published:** 2019-06-27

**Authors:** Igor Brikun, Deborah Nusskern, Diha Freije

**Affiliations:** 1 Euclid Diagnostics LLC, 9800 Connecticut Dr., Crown Point, IN 46307 USA; 2 Present Address: Luminex Corporation, 4088 Commercial Ave, Northbrook, IL 60062 USA

**Keywords:** Prostate cancer, PSA, Liquid biopsy, Circulating DNA, Biomarkers, DNA methylation

## Abstract

**Background:**

Prostate cancer diagnosis using the PSA test remains controversial because of overdiagnosis and overtreatment of potentially indolent cancers. There remains a need to increase the diagnostic lead time and to target treatment to patients with significant disease. One possible approach to overcome the limitations of PSA is to screen men for the molecular signature of early PCA, monitor the rate of disease progression and target treatment to patients who are likely to benefit from it. Such an approach requires a large panel of markers that define a molecular clock for PCA. We recently developed a panel of 19 markers for the non-invasive detection of PCA from urine DNA. It raised the possibility that additional methylation markers could be successfully analyzed from urine DNA, a prerequisite for increasing the diagnostic lead time and enabling disease monitoring.

**Methods:**

We developed semi-quantitative polymerase chain reaction assays for 13 additional markers and determined their methylation status in 150 urine DNAs from 94 patients with elevated PSA. Eighty five samples were obtained following DRE and 65 samples were from first void. We combined the data of the 13 new markers with the previously reported 19 markers and calculated the sensitivity, specificity, negative and positive predictive values at every threshold from one to 32 positive markers.

**Results:**

Using 10of32 positive markers as the threshold to recommend a biopsy yields a sensitivity of 81% (95% CI 0.68–0.93) and 93% (95% CI 0.84–1.02) and a specificity of 76% (95% CI 0.63–0.88) and 77% (95% CI 0.63–0.91) from DRE and FV DNA, respectively. The PPV was 71% and 77% and the NPV was 85% and 93% from DRE and FV, respectively.

**Conclusions:**

This study shows that large marker panels can be analyzed from urine DNA without loss of sensitivity or specificity. Using 32 markers improved the stratification of patients undergoing screening for PCA particularly for patients below the 10of32 threshold. The results show the utility of larger biomarker panels for PCA diagnosis and suggest that the development of the panels needed to monitor disease progression could be successfully accomplished.

**Electronic supplementary material:**

The online version of this article (10.1186/s40164-019-0137-x) contains supplementary material, which is available to authorized users.

## Background

Over the last 2 decades, prostate cancer (PCA) screening using the prostate specific antigen (PSA) test exposed the complexities and challenges associated with early screening for a common cancer [1]. PCA is a heterogeneous disease with a long natural history and a wide range of prognostic outcomes. PSA screening advanced the PCA diagnosis by 5 to 7 years [2, 3]. However, the improved lead time resulted in only a modest reduction in PCA-specific mortality and was not sufficient to diagnose all prostate cancers while curable [4].

Autopsy studies have shown that PCA can be histologically detected in approximately 50% of men over 80 years old, vastly exceeding its diagnostic and mortality rates [1, 2, 5–7]. The large reservoir of slow growing prostate tumors contributes to the overdiagnosis and overtreatment of PCA when asymptomatic men are screened with PSA. It brought into question the value of early screening [1, 3, 8–11]. Clinically significant disease which affects less than a quarter of PCA patients remains difficult to detect early [12, 13]. Its diagnosis is further complicated by a highly variable rate of disease progression. A number of non-invasive diagnostic and prognostic tests have been developed to improve on PSA screening but none have achieved the accuracy needed to safely reduce overtreatment [14–18]. Their primary objective is to predict the likelihood of a significant cancer in patients with elevated PSA. This approach is limited by the PSA test itself which lacks the specificity and lead time needed for an early cancer screening test. Non-invasive molecular tests to increase the diagnostic lead time and enable monitoring for disease progression are still needed. A longer lead time will ensure that all patients are diagnosed while their disease is curable. Measuring the rate of disease progression will enable the targeting of treatment to patients with high risk tumors before the cancer becomes disseminated. These tests will likely require large panels of markers that can be detected in circulating DNA and correlated with disease grade and stage.

The most common cancer-specific aberration is the progressive accumulation of DNA methylation. It affects hundreds of CpG islands and can be assessed in liquid biopsies. We recently published a biomarker panel for the non-invasive diagnosis of PCA [19]. The panel detects the cumulative methylation of 19 markers in urine DNA recovered following a digital rectal exam (DRE) or from first void (FV) samples. The test measures the number of methylated markers in urine DNA and shows that the likelihood of a positive biopsy increases with increasing number of methylated markers. Using six methylated markers out of nineteen (6of19) to define a cancer case yielded a sensitivity of ≥ 89%, a specificity of 71%, a positive predictive value (PPV) of ≥ 71% and a negative predictive value (NPV) of ≥ 90%. The ability to analyze 19 methylation markers from circulating DNA without loss of specificity was unexpected. It raised the possibility that larger methylation panels could be successfully analyzed from urine DNA without loss of diagnostic accuracy.

We undertook the current study to determine the feasibility of analyzing additional markers in urine DNA. We selected 13 CpG islands with methylation ranging from 50 to 80% in primary prostate tumors (FRZB, GPR147, GPR62, GRASP, HOXA11as, HOXBAS3, HOXCrcAS3, HOXD3c, HOXD4rc, HOXD8rc, KLK10, RASSF1, SLC16A5rc) (authors unpublished data) and determined their methylation in 150 DNAs from 94 patients using semi-quantitative methylation-specific PCR. In this study, we report the methylation of the 13 new markers as well as the cumulative methylation of the 32 markers.

## Methods

### Patient cohort

Urine samples were collected under an IRB protocol approved by Western Institutional Review Board (WIRB, study # 1139453, Puyallup, WA) and were previously described [19]. Patients with positive biopsies were grouped based on CAPRA risk criteria into a low risk group (CAPRA score ≤ 2) and an increased risk group (CAPRA scores ≥ 3) [20]. Four DNA samples were lost during processing reducing the number of DNAs analyzed to 85 from DRE and 65 from FV.

### Marker analysis

Marker assays were validated and urine DNAs were analyzed as described [19]. The MS-qPCR reactions were performed on the QuantStudio 6 (ABI) with the same bisulfite converted DNAs used for the first 19 markers [19]. Primer and probe sequences for all markers are shown in Additional file 1: Table S2. An assay for an imprinted gene (STIM1) was added to the primary amplification multiplexes to verify the recovery of amplifiable DNA from urine samples. The data for the additional 13 markers were collected after the first 19 markers were unblinded.

### Data collection

The data were tabulated using the software provided with the QuantStudio 6. The data were further transformed by subtracting the Cq values from 32 (except for the 0 data points) to generate an increasing range of values from 0 (no amplification) to 15 (highest level of amplification, lowest Cq) as previously described [19]. The data were used directly for statistical analysis with no further manipulations.

### Statistical analysis

Each subject in the study had at least one type of urine sample (DRE or FV) collected. Subject characteristics were summarized within the cases and within the controls, respectively. The cases are defined as the subjects with positive diagnosis of prostate cancer based on biopsy, and the controls are defined as those with negative diagnoses. Arithmetic mean and standard deviation were summarized for continuous characteristics, and frequency and percentage were calculated for categorical characteristics. Characteristics such as Gleason score and positive cores were available only for cases, and were summarized at both continuous and dichotomized levels.

The following statistical analyses were performed for both DRE and FV samples unless otherwise specified. Sensitivity and specificity associated with the presence of individual methylation markers were computed using the observed proportion of individuals with positive markers conditional upon diagnosis status, and their 95% confidence intervals were provided.

Similarly, sensitivity, specificity, negative and positive predictive value and their 95% confidence intervals were calculated for each of the possible number of positive markers among the 32 markers. The average methylation for each DNA sample was calculated by adding the values obtained for all 32 assays and dividing by 32. Box plots were generated by diagnosis status for both the number of positive markers and the average methylation levels of the 32 markers. ROC curves were plotted for the 32 markers based on either average methylation or the number of positive markers. We did not perform any modeling to further characterize the markers because of the small number of patients analyzed.

The average number of positive markers by patient grading group was compared using the Wilcoxon rank-sum test. Grading groups of grade 0 (negative biopsy), 1 (positive biopsy, low risk group based on CAPRA), or 2 (positive biopsy, elevated risk group based on CAPRA) and the group combining grade 1 and 2 (all positive biopsies) were considered. Similar analysis was performed to compare the means of average methylation by grading group. Paired sample analysis was also performed using the paired t test. All statistical analyses were performed using R version 3.4.4 (https://cran.r-project.org), and the AUC calculations were performed with R package “ROCR” with version 1.0.7.

## Results

### Patient characteristics

The patient cohort was described previously and included 52 patients with negative biopsies (controls) and 42 patients with positive biopsies (cases) [19]. The first void and DRE urine samples were collected prospectively up to 6 weeks prior to biopsy. Patient demographics are shown in Table 1. The mean Gleason score was 7.1 (SD = 1.3) and the mean number of positive cores was 4.2 (SD = 3.6). Clinical follow up was available for a subset of patients after the marker data was collected. Three patients who had a negative biopsy after urine collection were diagnosed with PCA within 2 years. They were included in the cancer group at the start of the statistical analysis.

**Table 1:** Patient demographics summarized for the overall population and by biopsy diagnosis

Variable	Cases (n = 42)	Controls (n = 52)
Age		
n (%)	42 (100.0%)	50 (96.2%)
Median	66	64
Mean (SD)	67.1 (7.1)	63.9 (7.6)
Range	48–84	50–83
PSA		
n (%)	40 (95%)	51 (98.1%)
Median	6.4	5.2
Mean (SD)	7.1 (3.3)	5.6 (2.7)
Range	3.26–18.92	0.63–14.9
Race		
Alaskan Native	1 (2.4%)	0 (0.0%)
Asian	0 (0.0%)	1 (1.9%)
Black	4 (9.5%)	5 (9.6%)
Hispanic	1 (2.4%)	1 (1.9%)
White	36 (85.7%)	44 (84.6%)
Missing	0 (0.0%)	1 (1.9%)
Urine samples		
DRE and FV	28 (66.7%)	32 (61.5%)
DRE only	10 (23.8%)	17 (32.7%)
FV only	4 (9.5%)	3 (5.8%)
Gleason score		
n	39 (92.9%)	NA
Mean (SD)	7.1 (1.3)	
≤ 7	26 (61.9%)	
> 7	13 (30.9%)	
Missing	3 (7.1%)	
Positive cores		
n	39 (92.9%)	NA
Mean (SD)	4.2 (3.6)	
≤ 3	19 (45.2%)	
> 3	20 (47.6%)	
Missing	3 (7.1%)	

The mean PSA for cases was calculated after excluding 2 outliers which were greater than 2X the highest remaining PSA value from cases. NA: not applicable

### DNA methylation in DRE and FV DNA

A presence (> 0) or absence of methylation (= 0) was used for the calculations of sensitivity and specificity regardless of the amount of methylation detected in urine DNA to limit any subjective interpretation of data to the analytical assay conditions. A low level of methylation may reflect a small number of methylated copies in urine DNA or may be an analytical error from incomplete deamination of genomic DNA (false positives). Including all positive markers regardless of level of methylation and without using cutoffs based on clinical data incorporates all potential false positives in the results of individual markers and in the cumulative urine methylation calculations and eliminates false negatives.

The 13 markers were recovered with variable frequencies from both DRE and FV. The observed sensitivities of individual assays ranged from 19 to 87% while specificities ranged from 46 to 100%. Table 2 shows the sensitivity, specificity, positive and negative predictive values (PPV and NPV) for all 13 markers.

**Table 2:** The sensitivity, specificity, NPV and PPV for new markers measured in 85 DRE and 65 FV urine DNAs

Marker name	DNA type	No. Pos/No. cases	Sensitivity	95% CI	No. Neg/No. controls	Specificity	95% CI	PPV	NPV
HOXA11as	DRE	'10/36	0.28	(0.13, 0.42)	'43/49	0.88	(0.79, 0.97)	0.63	0.63
	FV	'16/30	0.53	(0.35, 0.71)	'29/35	0.83	(0.70, 0.95)	0.73	0.67
KLK10	DRE	'17/36	0.47	(0.31, 0.64)	'44/49	0.9	(0.81, 0.98)	0.77	0.7
	FV	'10/30	0.33	(0.16, 0.50)	'29/35	0.83	(0.70, 0.95)	0.62	0.59
GPR147	DRE	'18/36	0.5	(0.34, 0.66)	'41/49	0.84	(0.73, 0.94)	0.69	0.7
	FV	'15/30	0.5	(0.32, 0.68)	'24/35	0.69	(0.53, 0.84)	0.58	0.62
GPR62	DRE	'20/36	0.56	(0.39, 0.72)	'32/49	0.65	(0.52, 0.79)	0.54	0.67
	FV	'24/30	0.8	(0.66, 0.94)	'24/35	0.69	(0.53, 0.84)	0.69	0.8
HOXD4rc	DRE	'11/36	0.31	(0.16, 0.46)	'41/49	0.84	(0.73, 0.94)	0.58	0.63
	FV	'8/30	0.27	(0.11, 0.42)	'29/35	0.83	(0.70, 0.95)	0.57	0.57
HOXD3c	DRE	'23/36	0.64	(0.48, 0.80)	'31/49	0.63	(0.50, 0.77)	0.56	0.71
	FV	'26/30	0.87	(0.75, 0.99)	'16/35	0.46	(0.29, 0.62)	0.58	0.81
FRZB	DRE	'20/36	0.56	(0.39, 0.72)	'33/49	0.67	(0.54, 0.80)	0.55	0.68
	FV	'23/30	0.77	(0.62, 0.92)	'26/35	0.74	(0.60, 0.89)	0.72	0.79
GRASPrc	DRE	'14/36	0.39	(0.23, 0.55)	'42/49	0.86	(0.76, 0.96)	0.67	0.66
	FV	'13/30	0.43	(0.26, 0.61)	'31/35	0.89	(0.78, 0.99)	0.77	0.65
HOXBAS3	DRE	'16/36	0.44	(0.28, 0.61)	'41/49	0.84	(0.73, 0.94)	0.67	0.67
	FV	'14/30	0.47	(0.29, 0.65)	'32/35	0.91	(0.82, 1.01)	0.82	0.67
HOXCrcAS3	DRE	'7/36	0.19	(0.07, 0.32)	'48/49	0.98	(0.94, 1.02)	0.87	0.63
	FV	'6/30	0.2	(0.06, 0.34)	'35/35	1	(1.00, 1.00)	1	0.59
HOXD8rc	DRE	'8/36	0.22	(0.09, 0.36)	'45/49	0.92	(0.84, 1.00)	0.67	0.62
	FV	'10/30	0.33	(0.16, 0.50)	'33/35	0.94	(0.87, 1.02)	0.82	0.62
RASSF1	DRE	'22/36	0.61	(0.45, 0.77)	'33/49	0.67	(0.54, 0.80)	0.57	0.7
	FV	'22/30	0.73	(0.58, 0.89)	'26/35	0.74	(0.60, 0.89)	0.71	0.76
SLC16A5rc	DRE	'12/36	0.33	(0.18, 0.49)	'45/49	0.92	(0.84, 1.00)	0.75	0.65
	FV	'11/30	0.37	(0.19, 0.54)	'29/35	0.83	(0.70, 0.95)	0.65	0.61

### Cumulative methylation in DRE and FV urine DNA from biopsy positive and biopsy negative patients

Using the presence of methylation (> 0) to classify markers as positive, we calculated the number of positive markers for each DNA sample. The median number of methylated markers in cases was 16 in both DRE and FV (range: 3–31 in DRE and 6–31 in FV). The median number of methylated markers in controls was 5 in both DRE and FV (range: 0–17 for DRE and 0–18 for FV). Table 3 shows the sensitivity, specificity, PPV and NPV for the total number of methylated markers (*nof32*) at every threshold from 1 to 32. Using 10 out of 32 positive markers (*10of32*) as the threshold to refer a patient for biopsy yields observed sensitivities of 0.81 and 0.93 and specificities of 0.76 and 0.77 for DRE and FV, respectively. A specificity of ~ 70% is the target specificity for a PCA diagnostic test because the sampling errors of prostate biopsies can lead to a negative finding for up to a third of cancer patients [21–23]. The *10of32* threshold yields an estimated NPV of 0.85 and 0.93 and an estimated PPV of 0.71 and 0.77 for DRE and FV, respectively. The results of the 32 and 19 marker panels were comparable. Increasing the number of markers to 32 was not expected to improve the sensitivity or specificity of the 19 marker panel but it improved the stratification of patients, in particular patients who did not meet the threshold for a positive diagnosis.

**Table 3:** Predictive performance of the number of positive markers for every threshold from 1 to 32 positive markers in DRE and FV DNAs

No. Positive markers	Sample type	No. Pos/ No. cases	Sensitivity	95% CI	No. Neg/ No. controls	Specificity	95% CI	PPV	NPV
1of32	DRE	'36/36	1	(1.00, 1.00)	'5/49	0.1	(0.02, 0.19)	0.45	1
	FV	'30/30	1	(1.00, 1.00)	'2/35	0.06	(− 0.02, 0.13)	0.48	1
2of32	DRE	'36/36	1	(1.00, 1.00)	'9/49	0.18	(0.08, 0.29)	0.47	1
	FV	'30/30	1	(1.00, 1.00)	'5/35	0.14	(0.03, 0.26)	0.5	1
3of32	DRE	'36/36	1	(1.00, 1.00)	'14/49	0.29	(0.16, 0.41)	0.5	1
	FV	'30/30	1	(1.00, 1.00)	'7/35	0.2	(0.07, 0.33)	0.52	1
4of32	DRE	'34/36	0.94	(0.87, 1.02)	'14/49	0.29	(0.16, 0.41)	0.49	0.87
	FV	'30/30	1	(1.00, 1.00)	'9/35	0.26	(0.11, 0.40)	0.54	1
5of32	DRE	'34/36	0.94	(0.87, 1.02)	'19/49	0.39	(0.25, 0.52)	0.53	0.9
	FV	'30/30	1	(1.00, 1.00)	'13/35	0.37	(0.21, 0.53)	0.57	1
6of32	DRE	'34/36	0.94	(0.87, 1.02)	'27/49	0.55	(0.41, 0.69)	0.6	0.93
	FV	'30/30	1	(1.00, 1.00)	'16/35	0.46	(0.29, 0.62)	0.61	1
7of32	DRE	'33/36	0.92	(0.83, 1.01)	'29/49	0.59	(0.45, 0.73)	0.62	0.91
	FV	'29/30	0.97	(0.90, 1.03)	'18/35	0.51	(0.35, 0.68)	0.63	0.95
8of32	DRE	'32/36	0.89	(0.79, 0.99)	'32/49	0.65	(0.52, 0.79)	0.65	0.89
	FV	'28/30	0.93	(0.84, 1.02)	'23/35	0.66	(0.50, 0.81)	0.7	0.92
9of32	DRE	'30/36	0.83	(0.71, 0.96)	'33/49	0.67	(0.54, 0.80)	0.65	0.84
	FV	'28/30	0.93	(0.84, 1.02)	'25/35	0.71	(0.56, 0.86)	0.73	0.92
*10of32*	*DRE*	*'29/36*	*0.81*	*(0.68, 0.93)*	*'37/49*	*0.76*	*(0.63, 0.88)*	*0.71*	*0.85*
	*FV*	*'28/30*	*0.93*	*(0.84, 1.02)*	*'27/35*	*0.77*	*(0.63, 0.91)*	*0.77*	*0.93*
11of32	DRE	'29/36	0.81	(0.68, 0.93)	'38/49	0.78	(0.66, 0.89)	0.73	0.85
	FV	'26/30	0.87	(0.75, 0.99)	'28/35	0.8	(0.67, 0.93)	0.79	0.88
12of32	DRE	'28/36	0.78	(0.64, 0.91)	'40/49	0.82	(0.71, 0.92)	0.76	0.84
	FV	'24/30	0.8	(0.66, 0.94)	'30/35	0.86	(0.74, 0.97)	0.83	0.83
13of32	DRE	'26/36	0.72	(0.58, 0.87)	'41/49	0.84	(0.73, 0.94)	0.77	0.81
	FV	'23/30	0.77	(0.62, 0.92)	'30/35	0.86	(0.74, 0.97)	0.82	0.81
14of32	DRE	'24/36	0.67	(0.51, 0.82)	'44/49	0.9	(0.81, 0.98)	0.83	0.79
	FV	'23/30	0.77	(0.62, 0.92)	'31/35	0.89	(0.78, 0.99)	0.86	0.82
15of32	DRE	'22/36	0.61	(0.45, 0.77)	'47/49	0.96	(0.90, 1.01)	0.92	0.77
	FV	'19/30	0.63	(0.46, 0.81)	'32/35	0.91	(0.82, 1.01)	0.86	0.74
16of32	DRE	'20/36	0.56	(0.39, 0.72)	'47/49	0.96	(0.90, 1.01)	0.91	0.75
	FV	'15/30	0.5	(0.32, 0.68)	'34/35	0.97	(0.92, 1.03)	0.93	0.69
17of32	DRE	'17/36	0.47	(0.31, 0.64)	'48/49	0.98	(0.94, 1.02)	0.94	0.72
	FV	'13/30	0.43	(0.26, 0.61)	'34/35	0.97	(0.92, 1.03)	0.92	0.67
18of32	DRE	'12/36	0.33	(0.18, 0.49)	'49/49	1	(1.00, 1.00)	1	0.67
	FV	'13/30	0.43	(0.26, 0.61)	'34/35	0.97	(0.92, 1.03)	0.92	0.67
19of32	DRE	'10/36	0.28	(0.13, 0.42)	'49/49	1	(1.00, 1.00)	1	0.66
	FV	'12/30	0.4	(0.22, 0.58)	'35/35	1	(1.00, 1.00)	1	0.66
20of32	DRE	'9/36	0.25	(0.11, 0.39)	'49/49	1	(1.00, 1.00)	1	0.65
	FV	'8/30	0.27	(0.11, 0.42)	'35/35	1	(1.00, 1.00)	1	0.62
21of32	DRE	'8/36	0.22	(0.09, 0.36)	'49/49	1	(1.00, 1.00)	1	0.64
	FV	'7/30	0.23	(0.08, 0.38)	'35/35	1	(1.00, 1.00)	1	0.6
22of32	DRE	'8/36	0.22	(0.09, 0.36)	'49/49	1	(1.00, 1.00)	1	0.64
	FV	'5/30	0.17	(0.03, 0.30)	'35/35	1	(1.00, 1.00)	1	0.59
23of32	DRE	'7/36	0.19	(0.07, 0.32)	'49/49	1	(1.00, 1.00)	1	0.63
	FV	'5/30	0.17	(0.03, 0.30)	'35/35	1	(1.00, 1.00)	1	0.59
24of32	DRE	'7/36	0.19	(0.07, 0.32)	'49/49	1	(1.00, 1.00)	1	0.63
	FV	'3/30	0.1	(− 0.01, 0.21)	'35/35	1	(1.00, 1.00)	1	0.57
25of32	DRE	'7/36	0.19	(0.07, 0.32)	'49/49	1	(1.00, 1.00)	1	0.63
	FV	'2/30	0.07	(− 0.02, 0.16)	'35/35	1	(1.00, 1.00)	1	0.56
26of32	DRE	'7/36	0.19	(0.07, 0.32)	'49/49	1	(1.00, 1.00)	1	0.63
	FV	'2/30	0.07	(− 0.02, 0.16)	'35/35	1	(1.00, 1.00)	1	0.56
27of32	DRE	'3/36	0.08	(− 0.01, 0.17)	'49/49	1	(1.00, 1.00)	1	0.6
	FV	'2/30	0.07	(− 0.02, 0.16)	'35/35	1	(1.00, 1.00)	1	0.56
28of32	DRE	'2/36	0.06	(− 0.02, 0.13)	'49/49	1	(1.00, 1.00)	1	0.59
	FV	'2/30	0.07	(− 0.02, 0.16)	'35/35	1	(1.00, 1.00)	1	0.56
29of32	DRE	'2/36	0.06	(− 0.02, 0.13)	'49/49	1	(1.00, 1.00)	1	0.59
	FV	'2/30	0.07	(− 0.02, 0.16)	'35/35	1	(1.00, 1.00)	1	0.56
30of32	DRE	'2/36	0.06	(− 0.02, 0.13)	'49/49	1	(1.00, 1.00)	1	0.59
	FV	'2/30	0.07	(− 0.02, 0.16)	'35/35	1	(1.00, 1.00)	1	0.56
31of32	DRE	'1/36	0.03	(− 0.03, 0.08)	'49/49	1	(1.00, 1.00)	1	0.59
	FV	'2/30	0.07	(− 0.02, 0.16)	'35/35	1	(1.00, 1.00)	1	0.56
32of32	DRE	'0/36	0	(0.00, 0.00)	'49/49	1	(1.00, 1.00)	na	0.58
	FV	'0/30	0	(0.00, 0.00)	'35/35	1	(1.00, 1.00)	na	0.54

The 10of32 threshold to recommend a biopsy is shown in italic

**Fig. 1:**
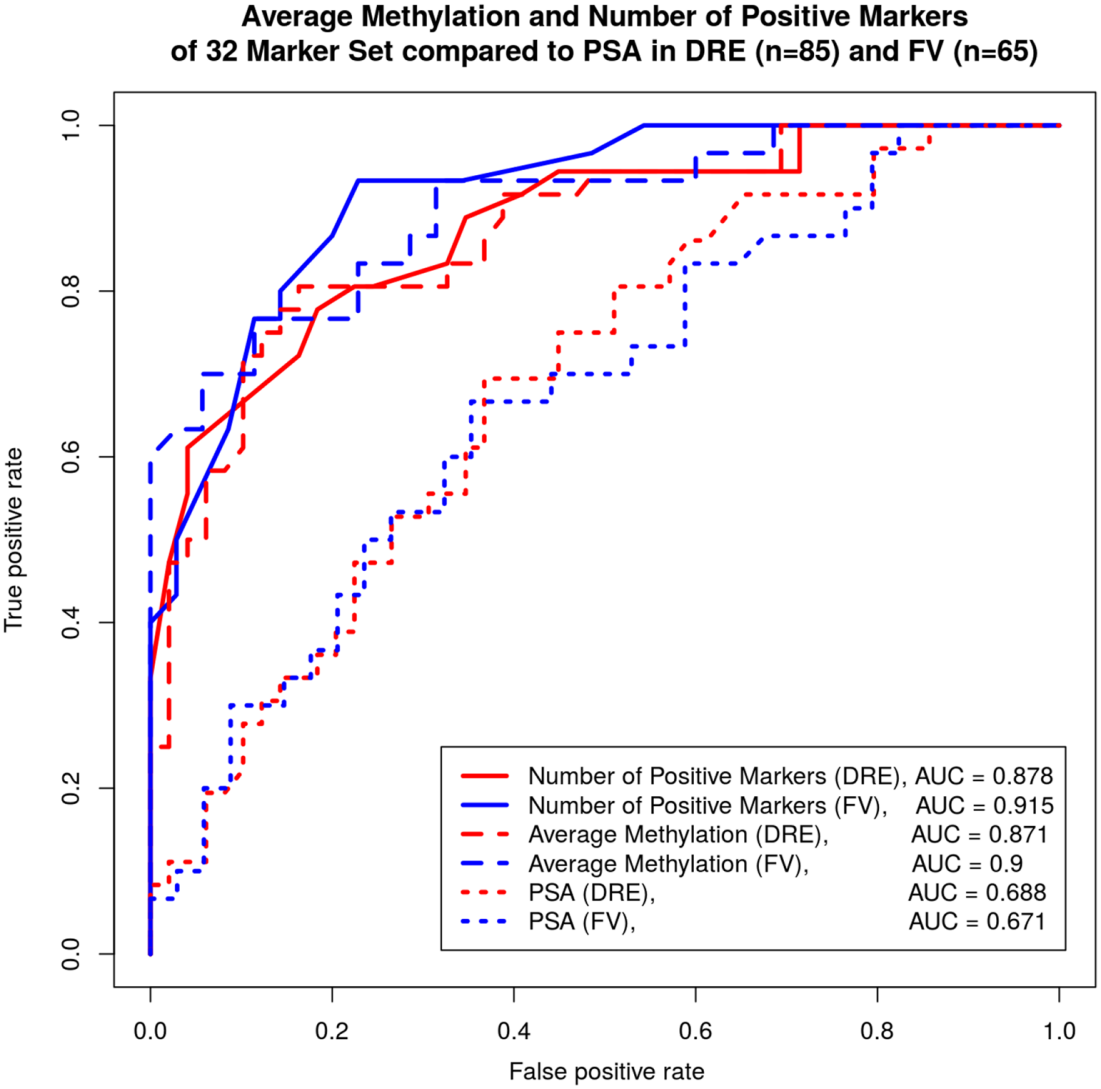
Receiver operating Characteristics (ROC) curves based on the number of methylated markers and their methylation levels generated for all 32 markers using the FV and DRE data. The ROC curves for PSA were also shown for comparison

The receiver operating characteristic curve (ROC) was calculated based on the number of methylated markers and the average methylation of all 32 markers. Figure 1 shows the ROC curves for the *nof32*, average methylation and PSA for the DRE and the FV data. The Area under the ROC curve (AUC) ranged between 0.87 in DRE to 0.91 in FV, a significant improvement over PSA (AUC = 0.688).

### Comparison between the DRE and FV methylation results

**Fig. 2:**
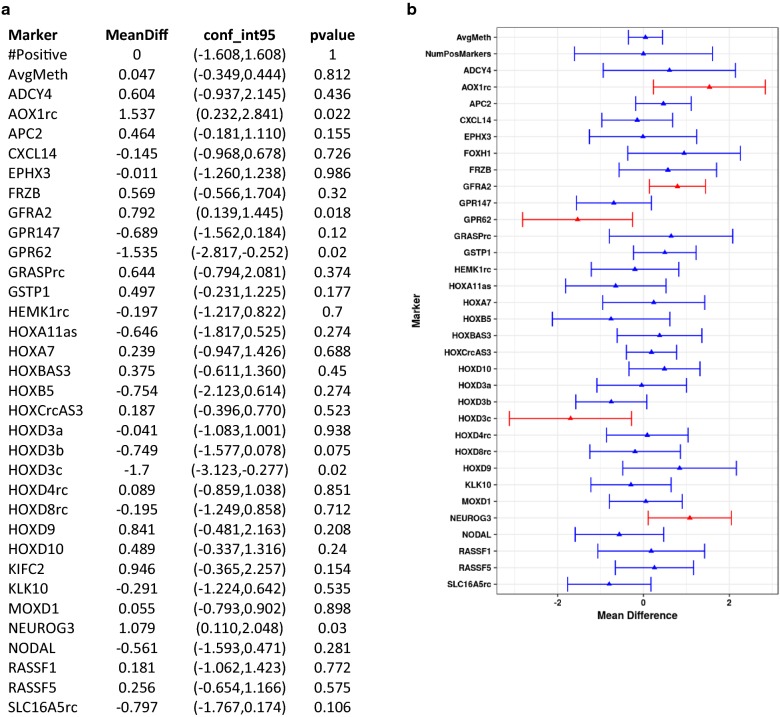
Paired-sample analysis: Paired test of methylation of individual markers between DRE and FV within the same case (N = 58). Panel **a** gives the values for mean difference, confidence intervals and p values for each marker. Panel **b** is a graphical illustration of the same data. Markers which showed potential difference (p < 0.05) between DRE and FV are shown in red and markers which did not (p > 0.05) are shown in blue

Both DRE and FV urine samples were available for 32 patients with a negative biopsy and 26 patients with a positive biopsy. Paired sample analysis was performed to compare the methylation of individual markers in DRE and FV DNAs. Figure 2 shows the observed within subject mean difference in methylation levels for individual markers, total number of positive markers and average methylation. There was no significant difference in the recovery of the majority of markers between DRE and FV. Of the 32 markers, AOX1, GFRA2, and NEUROG3 were better recovered from DRE samples (p < 0.05), and GPR62 and HOXD3c were better recovered from FV DNA (p < 0.05). The observed differences for these five markers are small. They are likely due to the position of the underlying assays within the CpG island and the small number of samples analyzed.

### Comparison of urine DNA methylation and Gleason score and tumor volume

**Fig. 3:**
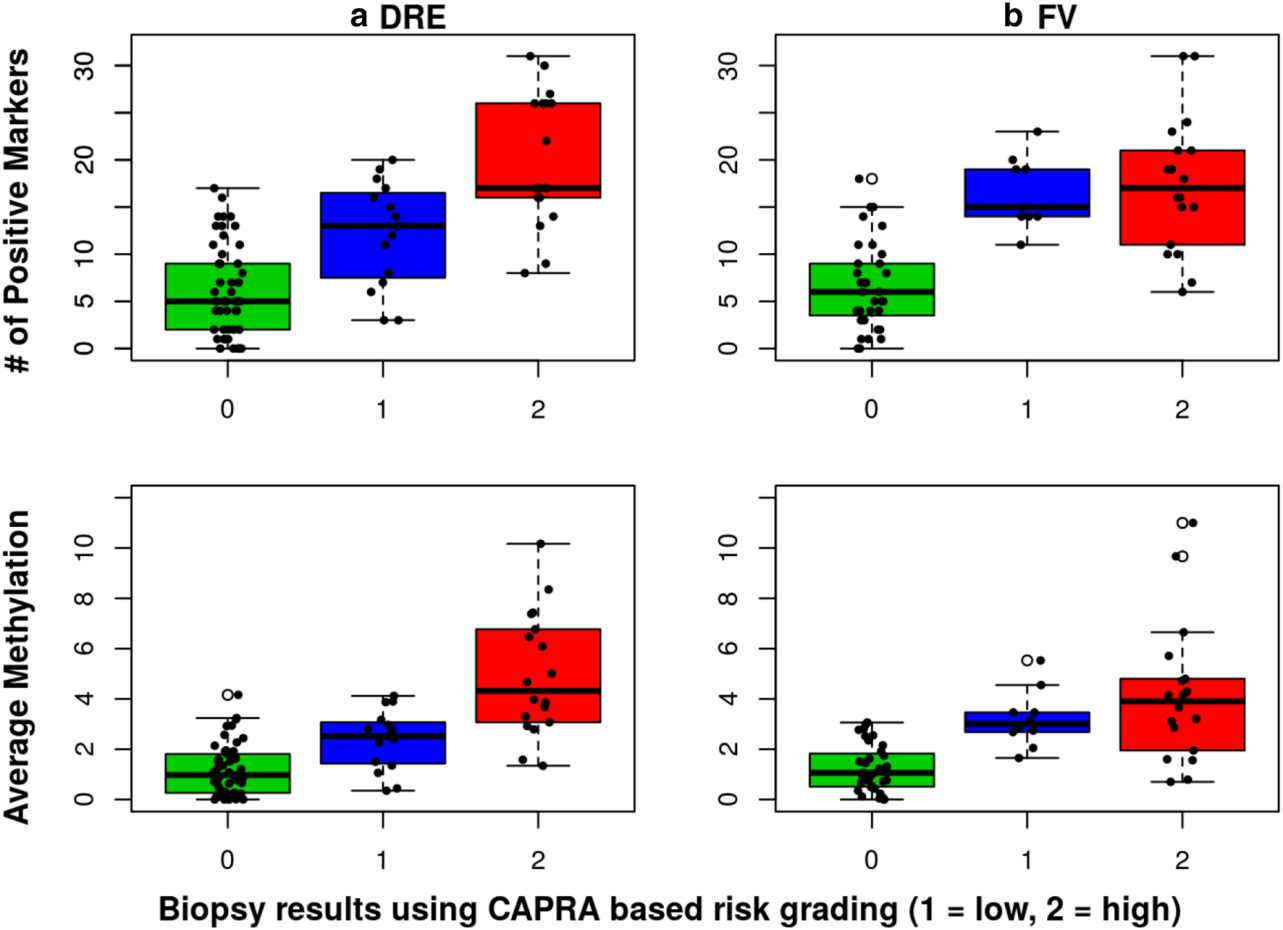
BOX Plot of number of methylated markers and average methylation levels. The distribution of the average methylation and the number of methylated markers is shown in green for patients with negative biopsies (Group 0), in blue for low-risk patients (Group 1) and in red for elevated-risk patients (Group 2). **a** The results of DRE samples and **b** the results of FV samples. The line inside each box indicates the median and the lower and upper hinges correspond to the first and third quartiles. The number of patients with DRE data was 49 for Group 0 (negative biopsies), 15 for Group 1, and 18 for Group 2. The number of patients with FV data was 35 for Group 0, 10 for Group 1, and 18 for Group 2. The mean, median, 1st and 3rd quartile, and maximum values obtained for the average methylation and the number of methylated markers are shown in Additional file 2

The histopathological findings (Gleason score, # of positive cores, tumor volume) varied widely between patients. Some cancers were limited to a single focus in a single core (GS of 6 to 8, ≤ 1% core volume) while others showed widespread, poorly differentiated cancer in multiple cores (GS of 8 to 10, up to 12 positive cores and up to 100% tumor volume per core). Similarly, the number of methylated markers and the average methylation varied widely. Patients with positive biopsies were grouped based on UCSF-CAPRA risk scoring system into a low risk group (Group 1: CAPRA score of 1 and 2) and an elevated risk group (Group 2: CAPRA score ≥ 3) [20]. Patients in Group 2 have an intermediate risk (CAPRA score of 3–5) except for 5 patients diagnosed with higher grade tumors (CAPRA score 6–9). The minimum, mean and maximum values obtained for the number of methylated markers and average methylation for each group are shown in Additional file 2: Table S3. The mean number of methylated markers and the average methylation differed significantly between cases and controls for both DRE and FV (Wilcoxon p-values < 0.001 for both DNA types). Furthermore, both parameters differed significantly between Group 1 and 2 patients for DRE DNA (Wilcoxon p-values of 0.001 for average methylation and 0.005 for the number of methylated markers) but not for FV DNA (Wilcoxon p-values of 0.4578 and 0.647, respectively). Figure 3 shows the distribution of average urine DNA methylation and the number of positive markers for all 3 groups. Methylation of DRE DNA outperformed that of FV in identifying patients with higher Gleason score or higher overall tumor volume.

## Discussion

We previously analyzed this patient cohort with 19 markers [19]. In this study, we determine the methylation of 13 additional markers and calculate the cumulative methylation of the full 32 marker panel. A single cutoff of 10of32 positive markers was used as the threshold needed to recommend a biopsy. However in a clinical setting, a personalized recommendation would be made based on the pattern of urine methylation for each patient. The 32-marker panel improved patient stratification over what was achieved with the 19-marker panel, especially for patients who did not meet the threshold needed to recommend a biopsy. Improving the stratification of patients undergoing PCA screening is important to decrease the frequency of repeat biopsies and to better predict the disease stage and grade in positive patients.

Methylation affects hundreds if not thousands of CpG islands in prostate cancer. The markers were selected strictly based on the analytical conditions used for the bisulfite conversions. In urine DNA, some markers like HOXD3, HOXA7, GPR62 and KLK10 were detected with comparable frequency from all cancer patients and are candidates for biomarker panels used for the early detection of PCA. Others like HOXD8rc, CXCL14, SLC16A5rc, and GRASP were recovered more frequently from patients with higher Gleason grade and higher tumor volume making them potential candidates for predictive or prognostic panels and for disease monitoring. This raises the question of how many markers are optimal for a diagnostic test and which markers to include in the final panel. Molecular tests are more expensive than PSA by a factor of 20 to > 100. Their successful clinical adoption requires maximizing their clinical utility and value to reduce the overall cost of PCA screening. Smaller panels or randomly chosen markers may not be the best choice for a clinical test. Markers need to be selected based on the purpose of the test, with markers methylated early in carcinogenesis used for a diagnostic panel and markers methylated in higher Gleason grade tumors used for disease monitoring. This panel can be readily modified to include markers that are associated with Gleason grade or clinical outcome. Additional markers could be used in conjunction with all or a subset of the markers presented here to generate diagnostic panels for different clinical purposes. The analysis of additional markers was performed to determine if increasing the number of markers has a negative impact on clinical sensitivity and specificity which was not the case. The results presented here make it more likely that much larger panels could be successfully used for PCA diagnosis from urine DNA. This study was not performed to finalize the marker panel for a clinical test but to determine the feasibility of analyzing larger marker panels. Prostate cancer detected on biopsy is highly heterogeneous in volume and Gleason grade with prognostic outcomes that are difficult to predict. To identify accurate prognostic markers will require the elucidation of the epigenetic profile of urine DNA from thousands of patients, an n significantly larger than the few hundred patients needed to validate the 32 marker panel. It will be important to plan the clinical trials accordingly in order to reduce the time and cost associated with marker validation.

For the 58 patients with both DRE and FV samples, paired sample analysis showed no significant difference in outcome between the 2 DNA sources. Overall, the methylation of FV DNA slightly outperformed that of DRE DNA at the 10of32 threshold, similar to what was obtained with the 19 markers. This supports its use for early PCA diagnostic tests. The small difference between DRE and FV could be attributed to the relatively small number of samples analyzed. The main advantage of using the FV samples for a screening test is the simplicity of obtaining multiple samples from each patient which reduces the sampling error rate and increases confidence in the diagnostic outcome.

The methylation of DRE DNA outperformed that of FV DNA in identifying patients with higher Gleason grade and larger volume tumors supporting its use for predictive and prognostic tests as well as tests aimed at monitoring disease progression. It is likely that the enrichment of cancer DNA following DRE contributed to the improved recovery of the assays used to determine marker methylation. The composition of the FV urine samples is dependent on the steady state release of tumor DNA into circulation and is subject to degradation and dilution. The FV DNA sampling error rate is expected to be higher than that of the DRE DNA. A larger marker panel, different markers or different assays for current markers may be needed to enable improved tumor profiling from FV DNA. However, it is likely that DRE DNA may always outperform FV DNA when extensive tumor molecular profiling is required.

We did not use PSA or age for the statistical analysis because the main objective was to determine the utility of urine DNA methylation as a standalone marker. PSA was used in preliminary modeling with the 19 marker panel. It did not improve the diagnostic accuracy of the panel because of the small number of patients. PSA may prove more valuable when a larger patient cohort is analyzed with more markers. For an early PCA diagnostic test aimed at increasing the diagnostic lead time, age will be an important variable. A risk of significant disease for a 68 year old patient with 15 positive markers out of 32 will likely be significantly different than that of a 50 year old with the same methylation profile. The first patient may only need to be monitored while the second patient will likely require treatment. For this reason, the clinical trials need to be designed to include age as a variable.

A correlation of urine and biopsy methylation will be a necessary step in the validation of individual markers to better understand their clinical utility. In this study, we did not compare urine and biopsy DNA methylation because pathology samples were not available. The primary indicators of disease severity (Gleason grade, the number of positive biopsy cores and tumor volume) vary widely at diagnosis, making a correlation between urine and biopsy methylation challenging. It is further complicated by the presence of methylation in benign prostatic tissues of PCA patients. We have previously shown that non cancer-adjacent, histologically benign tissues harbor extensive methylation similar to that observed in cancer cores [24]. It is unclear how much DNA the abnormal prostatic tissues contribute to cfDNA. We anticipate that at least some of the cfDNA is derived from non-cancer tissues. To accurately interpret the liquid biopsy results, all available biopsy cores regardless of histological findings should be included in the marker validation. To maximize the utility of the urine test, the number of patients needed for the clinical trials will likely be significantly higher than what is needed to validate the urine marker panel for a simple diagnostic test with a binary outcome. Long-term longitudinal studies will enable the quantitation of risks associated not only with the urine and tumor methylation but also the methylation present at the time of biopsy in benign tissues, particularly for patients considering watchful waiting.

## Conclusion

The cumulative methylation of large marker panels in urine DNA can be used for PCA diagnosis without loss of sensitivity or specificity. It can also improve the stratification of patients with negative and positive biopsies. Both FV urine samples and those collected following a digital rectal exam are suitable DNA sources for methylation analysis. The results of the 32 marker panel increase the likelihood that larger panels could be used successfully to obtain a detailed molecular profile of the underlying tumor or to non-invasively monitor cancer progression in patients under active surveillance.

## Additional file

**Additional file 1.** List of CpG islands, their sequences and a list of primers and probes used to assay their methylation.

**Additional file 2.** The range of average methylation values and the number of methylated markers obtained from DRE and FV DNAs by grade.

**Additional file 3.** The file contains the marker data updated to include all 32 markers. The patients were sorted and assigned new numbers that are unrelated to the alphanumeric code used as the identifier when the clinical samples were collected.

## Data Availability

The dataset generated during the current study is included in Additional file 3. The codes assigned to patients by the clinics and associated demographics were modified to ensure patient anonymity. The Additional file does not contain any of the information needed to indirectly identify the patients based on demographics. The genomic location of the CpG islands and the primers used to assay for methylation are included in Additional file 1.

## Abbreviations

PCA: prostate cancer
PSA: prostate specific antigen
DRE: digital rectal exam
FV: first void
CpG: 5′-deoxycytosine-phosphate-deoxyguanosine-3′
AUC: area under curve
ROC: receiver operating characteristic
MS-qPCR: methylation-specific quantitative polymerase chain reaction
SD: standard deviation
nof32: number of positive markers out of 32 total markers analyzed
10of32: 10 positive markers out of 32 markers
cfDNA: cell-free DNA

## References

[1] Grossman DC, Curry SJ, Owens DK, Bibbins-Domingo K, Caughey AB, US Preventive Services Task Force (2018). Screening for prostate cancer: US Preventive Services Task Force Recommendation Statement. JAMA.

[2] Draisma G, Etzioni R, Tsodikov A, Mariotto A, Wever R, Gulati R (2009). Lead time and overdiagnosis in prostate-specific antigen screening: importance of methods and context. J Natl Cancer Inst.

[3] Finne P, Fallah M, Hakama M, Ciatto S, Hugosson J, de Koning H (2010). Lead-time in the European Randomised Study of Screening for Prostate Cancer. Eur J Cancer.

[4] Schröder FH, Hugosson J, Roobol MJ, Tammela TL, Zappa M, Nelen V (2014). Screening and prostate cancer mortality: results of the European Randomised Study of Screening for Prostate Cancer (ERSPC) at 13 years of follow-up. Lancet.

[5] Bell KJ, Del Mar C, Wright G, Dickinson J, Glasziou P (2015). Prevalence of incidental prostate cancer: a systematic review of autopsy studies. Int J Cancer.

[6] Haas GP, Delongchamps N, Brawley OW, Wang CY, de la Roza G (2008). The worldwide epidemiology of prostate cancer: perspectives from autopsy studies. Can J Urol.

[7] Jahn JL, Giovannucci EL, Stampfer MJ (2015). The high prevalence of undiagnosed prostate cancer at autopsy: implications for epidemiology and treatment of prostate cancer in the prostate-specific antigen-era. Int J Cancer.

[8] Pishgar F, Ebrahimi H, Moghaddam SS, Fitzmaurice C, Amini E (2018). Global, regional and national burden of prostate cancer, 1990 to 2015: results from the global burden of disease study 2015. J Urol.

[9] Loeb S, Bjurlin MA, Nicholson J, Tammela TL, Penson DF, Carter HB (2014). Overdiagnosis and overtreatment of prostate cancer. Eur Urol.

[10] Cooperberg MR, Broering JM, Carroll PR (2010). Time trends and local variation in primary treatment of localized prostate cancer. J Clin Oncol.

[11] Neppl-Huber C, Zappa M, Coebergh JW, Rapiti E, Rachtan J, Holleczek B (2012). Changes in incidence, survival and mortality of prostate cancer in Europe and the United States in the PSA era: additional diagnoses and avoided deaths. Ann Oncol.

[12] Chang AJ, Autio KA, Roach M, Scher HI (2014). High-risk prostate cancer: classification and therapy. Nat Rev Clin Oncol.

[13] Cooperberg MR, Carroll PR (2015). Trends in management for patients with localized prostate cancer, 1990–2013. JAMA.

[14] Alford AV, Brito JM, Yadav KK, Yadav SS, Tewari AK, Renzulli J (2017). The use of biomarkers in prostate cancer screening and treatment. Rev Urol.

[15] O’Reilly E, Tuzova AV, Walsh AL, Russell NM, O’Brien O, Kelly S (2019). epiCaPture: a urine DNA methylation test for early detection of aggressive prostate cancer. JCO Precis Oncol.

[16] Zhao F, Olkhov-Mitsel E, Kamdar S, Jeyapala R, Garcia J, Hurst R (2018). A urine-based DNA methylation assay, ProCUrE, to identify clinically significant prostate cancer. Clin Epigenetics.

[17] Tomlins SA, Day JR, Lonigro RJ, Hovelson DH, Siddiqui J, Kunju LP (2016). Urine TMPRSS2:ERG plus PCA3 for individualized prostate cancer risk assessment. Eur Urol.

[18] McKiernan J, Donovan MJ, Margolis E, Partin A, Carter B, Brown G (2018). A prospective adaptive utility trial to validate performance of a novel urine exosome gene expression assay to predict high-grade prostate cancer in patients with Prostate-specific Antigen 2–10 ng/ml at initial biopsy. Eur Urol.

[19] Brikun I, Nusskern D, Decatus A, Harvey E, Li L, Freije D (2018). A panel of DNA methylation markers for the detection of prostate cancer from FV and DRE urine DNA. Clin Epigenetics.

[20] Cooperberg MR, Broering JM, Carroll PR (2009). Risk assessment for prostate cancer metastasis and mortality at the time of diagnosis. J Natl Cancer Inst.

[21] Bjurlin MA, Wysock JS, Taneja SS (2014). Optimization of prostate biopsy: review of technique and complications. Urol Clin North Am.

[22] Campos-Fernandes JL, Bastien L, Nicolaiew N, Robert G, Terry S, Vacherot F (2009). Prostate cancer detection rate in patients with repeated extended 21-sample needle biopsy. Eur Urol.

[23] Zaytoun OM, Moussa AS, Gao T, Fareed K, Jones JS (2011). Office based transrectal saturation biopsy improves prostate cancer detection compared to extended biopsy in the repeat biopsy population. J Urol.

[24] Brikun I, Nusskern D, Gillen D, Lynn A, Murtagh D, Feczko J (2014). A panel of DNA methylation markers reveals extensive methylation in histologically benign prostate biopsy cores from cancer patients. Biomark Res.

