# Supplementary material for: An expanded biomarker panel for the detection of prostate cancer from urine DNA
Source: Exp Hematol Oncol. 2019 Jun 27;8:13. doi: 10.1186/s40164-019-0137-x (PMC6598372; doi:10.1186/s40164-019-0137-x)
Supplement: Supplementary file 1 — Additional file 1. List of CpG islands, their sequences and a list of primers and probes used to assay their methylation. [file 40164_2019_137_MOESM1_ESM.docx]

**Supplemental Table 1 (ST1): List of CpG islands.** The positions of the CpG islands with respect to transcripts were automatically called by a bioinformatics pipeline. Many of the CpG islands may be involved in the transcription of other genes or alternative transcripts of the same gene. For example, HOXC-AS3 is -358 to 191 relative to NM_017409.3 transcript of HOXC10 and the HOXD4 island is -219 to 580 relative to start of NP_055436.2. The assignment of CpG islands to specific genes is for positional purposes only.

| **Gene Symbol** | **Gene ID** | **CpG Length** | **Chr** | **Chr Begin** | **Chr End** | **RNA Id** | **Pos Rel to 1st RNA base** |
| --- | --- | --- | --- | --- | --- | --- | --- |
| HOXD8 | 3234 | 550 | 2 | 176128665 | 176129214 | NM_019558.3 | -1075 to -526 |
| HOXD4 | 3233 | 800 | 2 | 176151415 | 176152214 | NM_014621.2 | 30 to 829 |
| HOXD3 | 3232 | 1100 | 2 | 176159854 | 176160953 | XM_005246509.4 | -964 to 135 |
| FRZB | 2487 | 750 | 2 | 182866815 | 182867564 | NM_001463.3 | -794 to -45 |
| RASSF1 | 11186 | 850 | 3 | 50340315 | 50341164 | NM_007182.4 | -228 to 621 |
| GPR62 | 118442 | 1250 | 3 | 51955550 | 51956799 | NM_080865.3 | 236 to 1485 |
| HOXA11-AS | 221883 | 550 | 7 | 27187900 | 27188449 | NR_002795.2 | 2492 to 3041 |
| GPR147 | 64106 | 1050 | 10 | 70254921 | 70255970 | NM_022146.4 | 27724 to 28773 |
| GRASP | 160622 | 1550 | 12 | 52006560 | 52008109 | NM_181711.3 | -385 to 1164 |
| HOXC-AS3 | 100874365 | 550 | 12 | 53984902 | 53985451 | NR_047506.1 | 68 to 617 |
| HOXB-AS3 | 404266 | 800 | 17 | 48581880 | 48582679 | NR_110331.1 | -8540 to -7741 |
| SLC16A5 | 9121 | 800 | 17 | 75087653 | 75088452 | NM_004695.3 | -288 to 511 |
| KLK10 | 5655 | 1100 | 19 | 51018526 | 51019625 | XM_017026993.2 | -259 to 840 |

**Supplemental Table 2 (ST2):** **List of primers and probes.** ST2 shows the list of primers and probes used to assay CpG islands methylation. The bisulfite number refers to the length of bisulfite used for each marker. BNF and BNR markers were used for the primary multiplex amplification as described in Methods. F, R and probes were used for MS-qPCR assays. CF and CR are the primers used for the control amplifications to verify the recovery of DNA following bisulfite conversion and multiplex amplification.

| **Assay** | **Primers and Probes** | **Bisulfite** |
| --- | --- | --- |
| FRZB | BNF: GGAAGAGAAGGYTTGGGAGT BNR: TTTCTAAARCCAAAAACRAATTCCC | 14 min |
|  | F: AGAAGGYTTGGGAGTCGTAGG R: GCTCGACTCGACGCTATCTT |  |
|  | Probe: TCGCACTCGCACGACTTCGCA |  |
|  |  |  |
| GRASPrc | BNF: GGAGAGGGGTTGAGTAGATTTTG BNR: CCACCCCTAARCCCCCAAC | 14 min |
|  | F: GAGTAGATTTTGAAGGCGGGC R: GAACGARCTATACGCGACGCT |  |
|  | Probe: TTCGGATACGGCGAGCGCG |  |
|  |  |  |
| HOXBAS3 | BNF: TTTTTGTTGGYGGGGAGAAGGG BNR: CCCTCTAARCCTTTCTCTCCTC | 14 min |
|  | F: ATTTAGCGAGGGAGCGAAATC R: ARCCTTTCTCTCCTCCGCG |  |
|  | Probe: TCGGTAGGCGGAGCGCGTAG |  |
|  |  |  |
| HOXCrcAS3 | BNF: GGTTAGATTAATGGGATTTGAAAATGG BNR: AAAACTCCRATACCCCTACCC | 14 min |
|  | F: GATGATTTAGACGGYCGT R: CCCGAAACARCTACTAAATAACGCT |  |
|  | Probe: TCGGTAGGCGGAGCGCGTAG |  |
|  |  |  |
| HOXD8rc | BNF: GTTTTGAGGYGGGAAAAAGGGTT BNR: TCTACCCTCCRCACTCCTCC | 14 min |
|  | F: TAGGACGCGGTTTTCGG R: CCGAAAACCGCAACCG |  |
|  | Probe: TTCGGCGCGTAGCGAATTAGG |  |
|  |  |  |
| RASSF1 | BNF: GGGTGTTAGYTTTYGTAGYTTAATGAG BNR: ACTCTCCTCARCTCCTTCCC | 14 min |
|  | F: TCGGTTGGGYTCGTG TTTCGTT R: ATCGAARCCCGCCCTATARC |  |
|  | Probe: CCGRCCCGCGCTTACTAACGC |  |
|  |  |  |
| SLC16A5rc | BNF: GGGYTTGGYGGAGAGGTGGTTT BNR: CCCACACCRCCCTAARCCC | 14 min |
|  | F: CCCACACCRCCCTAARCCC R: TACCTARCCGCGACGCT |  |
|  | Probe: ATCCCGCGATCCTCGTCGCG |  |
|  |  |  |
| GPR147 | BNF: GTTGTTGTTG AAGAAGGYTA GTTAG BNR: TATTCTTCACRCTATCCTAACTACC | 42 min |
|  | F: GCGTAGACGG TGATTAG R: GCTCTAAACGCTACTACTAC |  |
|  | Probe: TGCGGCGCGTTGAGYTGTTCG |  |
|  |  |  |
| GPR62 | BNF: TGGAGGTGGGGGTATTGTT BNR: CCAACAARCCCAACRACATA | 42 min |
|  | F: GTAACGGCGCGTTGTTG R: CACGACGCACAAATACGCCAAA |  |
|  | Probe: TCGTGGTGTTGCGTACGTCGG |  |
|  |  |  |
| HOXA11as | BNF: GGAGAAAAGGTTGGGGTTGAG BNR: CCTTTTCCCTCATCCATCTTCAC | 42 min |
|  | F: GAAAGGCGTTTCGGGAGC R: CCATCTTCACGACAACGCC |  |
|  | Probe: TGTATCGAAGTCGAGTGTTCGGAGG |  |
|  |  |  |
| HOXD3c | BNF: TTAGGGATTAGAGAGGGGAGGT BNR: CCAATCACCTCAAAAAACCCCGCC | 42 min |
|  | F: CGGGTCGTTTGTTACGCGTT R: CTCARCTCTARCCAATCACCTC |  |
|  | Probe: TTGAGTTCGCGGAACGAGTTACGG |  |
|  |  |  |
| HOXD4rc | BNF: GGGAGYTGGGTAAGGTTTTT BNR: TTCCAACCCCCRAAACTCTACC | 42 min |
|  | F: GGAGCGGCGTAGTGATCG R: CCCGACTTCGATAAACAACCTTC |  |
|  | Probe: TCGCGTAGGTAGCGTCGAGTTAG |  |
|  |  |  |
| KLK10 | BNF: GGTAGTGTAAAATGGAGYGGG BNR: CCRCCCCACTCAAACCCTATC | 42 min |
|  | F: CGGCGTAAGAGGAGGA R: CGATTTCTACCACCCGAATCCT |  |
|  | Probe: AAGAACGCGGCGAAGAGTTTACGG |  |
|  |  |  |
| STIM | BNF: GGGGTAGGGTAGYTGTTGT BNR: ATCAAATCCCARCTTCCCTTTCC | 14 and 42 min |
|  | CF: TCGTCGTAGGYTTGAGTTATTTG CR: CCACCGAAAATCTCCAACGCCA |  |

**3-Sequences of CpG islands:**

>HOXD3(C)_CpGLength:1100

CGGGTCCTGCCCAGACCTGGAGTCGCACAGATCACGGCGGGCAGTGGCTCAGCGCCTAGG

CGGCTCCAGGCCTCGAAGGACCAGGTTGGGGTGCTCAGGGATCAGAGAGGGGAGGTCGCT

CTGGGTCCGGGTCGCCTGCTACGCGCCTTTTCTGTCTCAGAAGTGGCGGTGACTCGGCTG

CTGAGTCCGCGGAACGAGCCACGGAATGGTGGTGGTGGCGGGGTTTTCTGAGGTGACTGG

CCAGAGCTGAGAGTCGCGGCTTCCACCTTTGGGCCGGAGCGGGTCCTCGCCCTGGGAGGA

GCTGGGCGTCGGCCTCCGCGGCGGGGAGGCCGCCTTGCCGGGGTGCATGAGGCTGCGGAG

TACTCCGCGGGCCCGGGAAGCTAGGGGTACCCTCAGCCTCTGCTGCTCCACGGCAGTCTC

CAGAGACGCTTCTAAGAGAGGCAGTTTCTAAAATTTCCAGCTCCCGGACCAGTCTGGCGG

AAGGCCCAGCCAGGGTCAGAGGTCGTTGTGGGGAGAGACTCTCAACGCCCCCAACCCACT

GAGGGCGGCCAGGCCAAGACTGAGTCGGCCCGGAGCTGCGAAAATGTGTTCTTTCCTTCC

GCCCCACACCCATCCGCGTCTGCCCCAGGAATGGGGCCCAGGTCCCAAGCCTCCTGCGCC

CTTCCTTCCAGCCCCCAGGCTTGGCTGCGCTCCGGGACTGGGTGGCGTGAAAGTTTCAGC

CTCAATCAGTACAAGCTTCCCTCGGGGTCACGTGAACAAATATGCTTGCATTTGAAGGCA

GCGTCTGTATTTCCCGACTATGAGGGGGTTTCCGGGGCTCTCTCCAAATCCAGAAACGAC

CACGTTCCGCAAGCAAAACAAATCCCAAGCTCTGGGGGGCCTGGGAGGGCTGGGCAGAAA

CCCAGGAGTGGGTGGGGGCGCGGGTGGCTGCCGCTCTGGGCCCGAgagcggacgggcggg

cgggtggaccgatgggcgcgcagcgcaggcgAAGCCAGCTCGGGGACTACGAACTCGTTC

CTCCTGCGTTTATTGGTAGTTGAACCTCAGCCTGGTTCCGTTCTACCGGGAATTCCGTGT

GCTCGAGTATATGGCCGTGT

>RASSF1_CpGLength:850

TAAAATCAGCGTATTTTTACATATAAGCAGCCACCTCTGCTCATCTGTGGCCCAGATACG

AGTGGAGTGCGACAAGGGATAAACCATTTTCGCGCACTCTTCAGCGATGGGGCGAAAGTA

ACGGACCTAGTCCTCGGGAGCTGTCCCCGCCGACCCCCTCTGCCGCGACTTGACCCGCGG

CGACTGCGCTGCCCCTTGGCTGCCCCTTCCGCTCTCGTAGGCGCGCGGGGCCACTACTCA

CGCGCGCACTGCAGGCCTTTGCGCACGACGCCCCAGATGAAGTCGCCACAGAGGTCGCAC

CACGTGTGCGTGGCGGGCCCCGCGGGCTGGAAGCGGTGGCCACGGCCAGGGACCAGCTGC

CGTGTGGGGTTGCACGCGGTGCCCCGCGCGATGCGCAGCGCGTTGGCACGCTCCAGCCGG

GTGCGGCCCTTCCCAGCGCGCCCAGCGGGTGCCAGCTCCCGCAGCTCAATGAGCTCAGGC

TCCCCCGACATGGCCCGGTTGGGCCCGTGCTTCGCTGGCTTTGGGCGCTAGCAAGCGCGG

GCCGGGCGGGGCCACAGGGCGGGCCCCGACTTCAGCGCCTCCCCCAGGATCCAGACTGGG

CGGCGGGAAGGAGCTGAGGAGAGCCGCGCAATGGAAACCTGGGTGCAGGGACTGTGGGGC

CCGAAGGCGGGGCTGGGCGCGCTCTCGCAGAGCCCCCCCCGCCTTGCCCTTCCTTCCCTC

CTTCGTCCCCTCCTCACACCCCACCCCGGACGGCCACAACGACGGCGACCGCAAAGCACC

ACGCGGAGATACCCGTGTTTCTGGAGGCCAGCTTTACTGTGCTAGAGGAAGAGGGTCCCC

ACATCCGGCC

>GPR62_CpGLength:1250

AGCAAGGGACAAGAGGAGCAGAGGACAGGTGATGGAAATCCTGCAGCTTTAGGCTCCATT

CTGCCATCTACATCCCAGCGCAGGGTGAAGCCTGAGAGCCCAAATGGCCAACTCCACAGG

GCTGAACGCCTCAGAAGTCGCAGGCTCGTTGGGGTTGATCCTGGCAGCTGTCGTGGAGGT

GGGGGCACTGCTGGGCAACGGCGCGCTGCTGGTCGTGGTGCTGCGCACGCCGGGACTGCG

CGACGCGCTCTACCTGGCGCACCTGTGCGTCGTGGACCTGCTGGCGGCCGCCTCCATCAT

GCCGCTGGGCCTGCTGGCCGCACCGCCGCCCGGGCTGGGCCGCGTGCGCCTGGGCCCCGC

GCCATGCCGCGCCGCTCGCTTCCTCTCCGCCGCTCTGCTGCCGGCCTGCACGCTCGGGGT

GGCCGCACTTGGCCTGGCACGCTACCGCCTCATCGTGCACCCGCTGCGGCCAGGCTCGCG

GCCGCCGCCTGTGCTCGTGCTCACCGCCGTGTGGGCCGCGGCGGGACTGCTGGGCGCGCT

CTCCCTGCTCGGCACGCCGCCCGCACCGCCCCCTGCTCCTGCTCGCTGCTCGGTCCTGGC

TGGGGGCCTCGGGCCCTTCCGGCCGCTCTGGGCCCTGCTGGCCTTCGCGCTGCCCGCCCT

CCTGCTGCTCGGCGCCTACGGCGGCATCTTCGTGGTGGCGCGTCGCGCTGCCCTGAGGCC

CCCACGGCCGGCGCGCGGGTCCCGACTCCACTCGGACTCTCTGGATAGCCGCCTTTCCAT

CTTGCCGCCGCTCCGGCCTCGCCTGCCCGGGGGCAAGGCGGCCCTGGCCCCAGCGCTGGC

CGTGGGCCAATTTGCAGCCTGCTGGCTGCCTTATGGCTGCGCGTGCCTGGCGCCCGCAGC

GCGGGCCGCGGAAGCCGAAGCGGCTGTCACCTGGGTCGCCTACTCGGCCTTCGCGGCTCA

CCCCTTCCTGTACGGGCTGCTGCAGCGCCCCGTGCGCTTGGCACTGGGCCGCCTCTCTCG

CCGTGCACTGCCTGGACCTGTGCGGGCCTGCACTCCGCAAGCCTGGCACCCGCGGGCACT

CTTGCAATGCCTCCAGAGACCCCCAGAGGGCCCTGCCGTAGGCCCTTCTGAGGCTCCAGA

ACAGACCCCCGAGTTGGCAGGAGGGCGGAGCCCCGCATACCAGGGGCCACCTGAGAGTTC

TCTCTCCTGAGCAGGAGAAAGGAGGGTGGTTTCCGTGGGGGCTCATCCAA

>HOXA11-AS_CpGLength:550

GACGGGGTTGTGGTGGCCCGCCAGCGGGGGATGCCAGGCCACCTCCCCCAGCGGCACGCA

GCCCCTCTCTTAATTAGATCGGTTTTCCCCTGGTGTCCGGGAGAGCGGTCCCGGCAGAAA

GGTCGGTATGGGGGTGTGCGCTGTTCCGCATAACCACTGCCTCCCATGTCCTCCTCGAGG

GCCGAACCGAGAGGGTGCTGGCAGGGCTGGATCCCACGGGTGTCCGCAGGAGACAAAGGC

GAATTCCGGAGAAAAGGCTGGGGCTGAGAAAGGCGCTCCGGGAGCGGCTGGCAGGGCAAT

TCGGCAGGCTGCACCGAAGCCGAGTGCCCGGAGGGACTTGCCGCCCGGAAGGGGGTGTGT

GGGGGCGCTGCCGTGAAGATGGATGAGGGAAAAGGTTTTTGATATCAGCAGAAGGGAAAA

CGCCTGGAGTGGCCGAACACTTTTAGTTGCCCAGCAGGAATAGGAGACGGGTACTCAGCT

CCCCAAGGCTGCGCAATATCCCAGCTTTGCCCGCTCCTGCCCTCGTGTTCGGAATATGCT

GGCGGTGTGA

>HOXB-AS3_CpGLength:800

GGGTTTATGGGCGACTCCCAGGACGGGTTTCACCGGAGCGGGAGGCTGCCTCTCCCCTTC

ACCGCCCAGAAGGATACGAACAGCAACACGCATAATAGTAAAATAAGAGCAGGGGCTGCG

CGCGAGCTGAGCCACCTCCCCTGCGCTCCCGGGTCTTTCCGTGGCTTCCCAGCCGCCTTC

TCCTGGCAGCCACAGGTTGCTAGCGTGACCTCGCCCCTCCCAGCCACCCTCCGCCGTCCA

GGCGTGCCCAGTCCGCTAGGAATCATCACCTTTTGTTGGCGGGGAGAAGGGACTCAGCGA

GGGAGCGAAACCGAAGGCCCGAGCGGAGGCGAGTCCCCAGCCGGCCTGCGCACCGGCGGC

GGCGGCGCGGAGGAGAGAAAGGCCCAGAGGGGGCGCGGGCGAGGGTACTCACCGGGAGGC

TCCCCAgccccgccggccgccgagccgcccgcggacgcgccgggcAACAGCTGAGCTCTC

TCCTGTCCACTATTGTGTGTCCGGAAAACCGGACCGCGGCGGCGGACACAGCCACGGGCT

CCCTCAGAAGCCGGAACGTGACTTTTTTTTTTGGTGTGTTTTTGTTTTGGAAGCGCAAGT

TTTCGTGTTAATCTCATACTTTGGCAGTCTTTGTTAAACAGCTAGGCGCGTCACGAGGCT

CTCAAACATTTTAATAATTTTTAGACGCCGTGACCTGCGGTTCACCTCTCTCCCAGCCTC

CCCCACGAGCAGGCAGTTACTATAATATGCTCGCGCAGTTCGCTGGGTCATATTGAATTT

TGCGTAGGTCATTACTTGGG

>HOXC-AS3_CpGLength:550

ACCTCCCCTGCCGCCCGGGGGGCGCTTCCTTTGTTCGCGGGGAAGGGCTCCGGTGCCCCT

ACCCCGAGGCAGCTGCTAGATGGCGCTGTTACTCCACTCTGCGCGCTCCGCCTGCCGACA

ACTTGACCCCGCTGACGTCACGGCCGTCTGAATCATCAAGGCCATTTTCAAATCCCATTG

GTCTAGCCGTCACATGGTGAGAACCGAATGCGCGGATAATTACGGAGCTGATATTTcccc

ccctccccttctttttcctccctcccctccaaccgcgccccccctcccGGATGGGGAAAA

AAAAAGATGTCAGCTCCTCCGCTGTAGTATTGCTCCTTAAAAACCCCTCTCTCTGAAAAT

GACATGCCCTCGCAATGTAACTCCGAACTCGTACGCGGAGCCCTTGGCTGCGCCCGGCGG

AGGAGAGCGCTATAGCCGGAGCGCAGGCATGTATATGCAGTCTGGGAGTGACTTCAATTG

CGGGGTGATGAGGGGCTGCGGGCTCGCGCCCTCGCTCTCCAAGAGGGACGAGGGCAGCAG

CCCCAGCCTC

>GRASP_CpGLength:1550

CCCCGACTCCCCAGGCTTCTTACAGTGACCTCTTACCGTGCCCCACTCCATGAATCGCCA

GAGCTATTCGTCCCTAAATTTCAAACCTTGCGCAATGTCCCTTCACAGACCCCTCCAGGT

ATCACGCAGCCCCGAGCCCCGAGCCCCGCCCCGGGGGCCTCATCCCGCCCCTTCGCGTCC

GCGGCTCGTTTTCCCCCACTGAGCGCCCAGCTCCCGCAGTTTCCCCGGCCGTCGAGCGCC

GTGGGCGGGGCTCCAGGGCGGCGGCGCCTCGCGGGGAGGGTCCTCCGTGCTGGGGGCGAG

GCCACCCGAGGCAGCTCCCCGCCCGCCCCCAACCCCGCCCCGCTCTCGGAGCCTATAAAG

GGAGGCGACCCGCGGCCCGCCCGGCTGGCATCCCCCAGCCGCCGCCAGCCCCGCCGAGGG

GAGCCAGCGCCGTCTCTGAGGGGCGTCCGGCGCCGGAGCCATGACCCTCCGCCGACTCAG

GAAGCTGCAGCAGAAGGAGGAGGCGGCGGCCACCCCGGACCCCGCCGCCCGGACTCCCGA

CTCGGAAGTCGCGCCCGCCGCTCCGGTCCCGACCCCGGGACCCCCTGCCGCAGCCGCCAC

CCCTGGGCCCCCAGCGGACGAGCTGTACGCGGCGCTGGAGGACTATCACCCTGCCGAGCT

GTACCGCGCGCTCGCCGTGTCCGGGGGCACCCTGCCCCGCCGAAAGGTGCGTCCCCCGCC

CGCCTTCAGGATCTGCTCAGCCCCTCTCCGACTCCCTACAGGGCCTGCTGACTCCGCAGT

GCCCTCTCCTCGGCGTCCGCGGAGTCCCCCACCTTCTTCCCCGGCCCGCTGGGTGCCTCG

ACTCCCCGCGTTCCCCGCTGCTGCGAAGGCCGTGGCCCTCGCCTGCACACCGCGCCCAGG

CTCGGTGGCTCTTAACTCCGCGCCCCATGCACGCCCCCTCTCTCCCTCCTTGACTCCTCC

CAGCACCCCCCTTCTCCTACCCGCTCCATCTGGCTTTCTGCCCCCCATGCCCCGCCTCCC

CGTGGCCAGGTGTCCTGGGTCCCCAGGAGCCCCTCGCCCGAGGGACAGAGACAGCCCCAG

GCAAGTTGAAGGTCCGAGAGCCCCCGGTGGGAGAAGCGGGCCGGTGGCTGCGCCGCGTGC

GTTCTCACTCTGAGGAAGTGCGTGGGGAGCCGCTGACTCCGGATAGCACACCCTTCCGAG

GGGACTCCCCGATTCCTGGGCTGGGGGCCTGCCGCCTGGCCCCACGTCTGACGTACGGGG

CGCGAGGGCCACTGCTCCCTGGACTTCTGTCGGAACCGGACGCAGTGGGAGGGGTCGCAG

GGCGCCCGCGGGGCAGGAAGGATGCGGGCCGCGCCCACCTCTGAGTCCCCTCTGCCAGCC

TCTTCCTCTGGCCCCAGGAGACCTGAGGCTCAGAACCTACACAACACCAGGTTAAGAAGA

GGGGCCTGGTGGCCTTTCCTCACCCAGCCGCCCTCCTTCGCCCCGGCCCCCAGCTAGCCC

CCACACAATGAACAGCTTGTTGAGAATTTGCATTTTATGAAAATCATGTT

>NPFFR1(GPR147)_CpGLength:1050

GGCCCTGAGGCAGCGTCCCGCCCTCCCTGGACCCCCTCAGATATCCCAGGCTGGAATGGT

GAGGGGCAGGTGGGAGCAGCCAGGCCCTTCCCTGGGCAAGCCGTGGTGAGCCACCCGCCC

ATTCCGCAGCGGGAGGCGGCCGGGCCTGGGGGCCCCACTGCTAGGGCCCGACTCAGAGGG

CAGCCCGGAGTCGCTGGGCCGCACCACCACGAAGACCCGCCTGTGCAGAAGCCCGCCGGG

CCGCTCGGAGTAGGCCTCCTTGTGGCTCCCCGACGGGCGCGGGCAGAGGCGGGCGCGGAA

GGCGGCCTGGAAGCCGCGGCGGAAGTTCTCGTTGAAGTAGCCGTAGATGATGGGGTTGGC

GCTGCTGTTGAAGAAGGCCAGCCAGTGCGCGAAGGGGAAGGCGTAGACGGTGACCAGGTG

CAGCTGCGGCGCGCTGAGCTGCCCGTAGTCGATGAGCAGCAGCAGCGCCCAGAGCGGCAG

CCAGGACAGCGTGAAGAACAGCGCCACCATGACCAGCATGTGCACCACGCGCGCTCTGCG

CCGCGATGCTCGCGGGTCCGCAGCCTCCTcgcccccgggggccgggcccggggccTGGCA

GAGCTTGCGCGCGATGCGGGCGTACATGACCACGATGAGCGCCAGCGGCGCCAGGTAGAT

GTGCGAGAAGAGCACAGTGGTGTAGACCCTGCGCATGCCCTTCTCGGGCCAGGCCTCCCA

GCAGGAGTAGAGCGGGTAGGAGCGGTTGCGGGCGTCCACCATGAAGTGGTGCTCCTCACG

GGTGACGGTCAGCGTGACGGCCGAGGGACACATGATGAGCAGCGCCAGGGCCCAGATGAC

GGCGATGGTGACGAGCGCCTTCCGCAGGGTCAGCTTCTCGCGGAAAGGGTGCACGATGCA

GCGGAACCTGCCGCGGGGAGAGAGACAGGCGGGATCTGGGTGGGTCCTAGGGCCCCTGCG

AGGGGACGGTGGGTGGGATGCGGGCACCTGACCTTCATCATCGCATCTAGGGCGGCGTCG

AAGAACAGCTCAGACCTGAATTGGCTGAGT

>KLK10_CpGLength:1100

aatcccatctctactaaaaatacaaaaaactagccgggcttggtggcgggcgcctgtagt

cccagctactcgggaggctgaggcaggagaatggtgtgaacccgggaggcggagcttgca

gtgagccgagatcgcgtcactgcactccagcctgggcgacagagcgagactccgtctcaa

acaaacaaacaaacaaaACAAAACACGGTAGTGTAAAATGGAGCGGGGCCGGGATGGGGC

GGCGTAAGAGGAGGAGAAAGAACGCGGCGAAGAGTCCACGGAAGAGCGAGGATCCGGGTG

GCAGAAATCGGACAGGGCCTGAGTGGGGCGGGTCACCCAGGGGCCGAGCCAGAAGAAGGG

CCCAGCTGACTTGGGGGCGGGCCGTGCTCCGGAGCGCTGGGTGGGTGCTGGGGTCTCGGG

GGAGGAGAGGTGCGCGGGGCTTGGTGACGGGAACACATTCTCCTCCCGCCCGTGCCTCCC

ACCGGCGCCTCTCCCCGCCCCCTGCCCCCGACCTTACCCCAGAGTTGCGCCATCAGCAGC

GGCAGCAGCTTCGCCAGAGCCCGGGCGCCAGAGGCGGCGGAGAGGTGGAGGTGCGGAGCT

CTCATGGCCAGGATCTGCTGGGGTGTGTGCAGGGGCGGGTTAAAACAGATGCTCCGTTAG

AGACCCCCACCTCGCCGCGCTCATCCGCCCAGCCTGGGCCACCCCAGCCCGCAAGCACCC

TTTTGACCTGCAGCCGATAACCCCAGGGGCTGGCAGACGGGAGATTCGGGCTGGAACAGC

GGTAATGGGCACAATTACCCTAATGACGCCCCTCGCGGCATCTTCCCGTCCTCCCTGTGC

CCGAGTGGAGCGCTCTCCGCGCCCCAGCTACCCTGGCTGCAGCCACGCCGCGCCCGAGGT

TTCCCCCTCCTTCACGCGCGGGGTGGGGATCCGAGGCTCGGAGCCAGTGGGAGCCTCTTT

CTCAACCTCACAGCGGGGGGACTTCCGCGTCCCGCAGGTGGAGAAACCGAGGCTCTAAGC

CGGCTCCTGCCTGTGGCCCGGGGGTCCCCACCCATCCTCTCGCAATCCACCCCAACCCGG

GTGGGGTGCAGGTAGCTTCA

>SLC16A5_CpGLength:800

GGGAGGTGTGGTCTCTTTATGGCATGTTATCTCCGCCCCAAGCCCGGGGCCCTAGACCCT

GAGCCGGGAAAACCAGAAGCTAGAAAGAGGGCATCCGGGTTGTGCAAATTTCAGACACAC

GGATATCCCGTGGCGCCTGCGTCCGGAGCCCGGCCCTGCTGCTGGACTGTCCCCGGCCAC

GCCCGCCGCCCCTCCCGCATCCTGGCCCCTTCCCCACCCGACGGAATCCCGGGGAGGCTG

GAAGGTGGGTCCCCGCCCACACCGCCCTGGGCCCGCCCTGCCTGGCCGCGGCGCTTGGCC

CAGTCCCGCGGTCCTCGTCGCGACCGGCCCGGGCCGCAGGACGCCGCGCTCGGGGGAGCT

GAGCCACCTCTCCGCCAGGCCCGTGCGAGCGGGTGAGCGGGCCCCCCGGGCCCCACTGCC

TCGGACGCGTCCCCTCCTCGCCCAGTCCCCTCTAGCGCTGTCGCAGACCTCCTGCCAGGG

CCCCAGGTCTCCTCCCGCCTGGGCGGGCTCAGTGGCGGTGAGGATCTAGAAAAGTCTTCC

TGTCCTGGTTCTGGGCTGCCCCGGGGCCTGGGGTGATGACCCGCAAGCCCAAGGGCGCTC

GGACTCCTGGCCCGAAATTCCAGGGCGGACACAGCCCGGGATCCCAGCAGCCCCAACTCG

GAGGAGCTCTGCCCCGGGGAGGGAGGGACAGCGGAGTGGAAACCAGATTCCGGGGCAGCA

CTGCGGGGATGGGCAGGGCGAGAGTACGGCATGAGGTCCCAGTTGCCTGTCCCAAACCCT

TGCTGGTTTTGCAGTGAATA

>HOXD4_CpGLength:800

GCCATAGCAAGCTACTTGATTACACGTATGTTATTTAGTTAAATTTGTGAAAATTATGAG

ATGCTCACCAACCCGGTGATAAACTTGCTCCCTCGCCATTGGCTGGCCTGGTCACATGGC

TGCCCAACTTTATTCAGTTGACAGCAAGTAGGAGGGCCCTATGGAAGGAGAAAAAAAGAC

AACACGAGAAAAATTAGTATTTTCTACCTTCTGAAATTAATGGTCATGAGTTCGTATATG

GTGAACTCCAAGTATGTGGACCCCAAGTTCCCTCCGTGCGAGGAGTATTTGCAGGGCGGC

TACCTAGGCGAGCAGGGCGCCGACTACTACGGCGGCGGCGCGCAGGGCGCAGACTTCCAG

CCCCCGGGGCTCTACCCACGGCCCGACTTCGGTGAGCAGCCTTTCGGAGGCAGCGGCCCC

GGGCCTGGCTCGGCGCTGCCTGCGCGGGGTCACGGACAAGAGCCAGGCGGCCCCGGCGGT

CACTACGCCGCTCCAGGAGAGCCTTGCCCAGCTcccccggcgcctccgccggcgcccctg

cctggcgcccgggccTACAGTCAGTCCGACCCCAAGCAGCCGCCCTCCGGGACGGCACTC

AAGCAGCCGGCCGTGGTCTACCCCTGGATGAAGAAGGTGCACGTGAATTCGGGTAAGGCT

AGGGTCCAGTAACCTTTCTGTCCACATCCCAGCCCGTTAGCCTGGGTCCTCTGGAAGGGG

GTGCGAGTAGGTGGGGGCGTGTGGAGCTTCCATGGGCGCCGCAATTACTCTCCCCATAAA

TTTTTATAGCTGAGGGAGCA

>HOXD8_CpGLength:550

GACTGGGCCACATGACTCGGGCAACTTTTCTTCATCTCTGCTCACAAACTAGGCTGAGCC

TTCTCTTTCTCTGCTTTGGGAAAAATTCGTAACTTTGAGGCCTGCGCGCTCTCTCGGCAG

TAATTTCACAGGCTTGGCGGAGGAAGTAGGATCGTTAGCATAAGATGGGCCGCCTGCAGC

TGCCTGGAACCGGCGCGATCTGAACGCCGGCTGGGAGGCTCCTGGGGGGAACTTGCGGTC

GTCTGCCCTCCGCACTCCTCCGGGAACCGCAGCCGGCCCTGGTTCGCTGCGCGCCGGGGC

TGAGACCCGGGAGCCGCGTCCTGCCCGAGGAAATGTCACCCTCCCCAGCGCGAGCCCTTT

TTCCCGCCTCAGAACGTTTCTGTCCGCTCTTCTATTTACTCTCTCAGCAAGCCTAATGCC

CCCTCCTGCATTCTTCAGCCTCCCCCTGCGCCCAGGGCTGACGTCTATCAAGGGTGAAAT

GATGGAAACTATATTCATGGGCATGATTTCCATTAAATATCAATTAACCTGAGAGCCTCG

GCCAGGCTTG

>FRZB_CpGLength:750

CTGGCAGAAGCATCAGACTTCGCAGATCAGTCAGATAAGAGGGACACGAGAGGAGGCGAG

GAAGACATAAAAACAAAAGTAGAATTCAGCTGAGAGCTAGTTGGAGCTCTGGGGTGTCTC

CTCCCCTAGATCACGCACACAAAAAGATGCTAAAGAAAGCATTAAATTCCCCAAAGTGAG

GAGGACAGAGGGGTTGGGGGGAAGGAGGGAGGAAAAGCTCCTAATGGGAGAGAAGGGTGC

AGTAAGAGTTTACAAGTCCTGTGTGGGCTAAGAGCTGCGGCCGCCGCTGCGGCTGCGACC

CTGGCTGGCAGGAGGGACGCTCAGGAGGAGCCCCGCAGGGACGTCTGTGCCTCTGCCCGG

GCGGCTCTGCACTTTCCTACCTCCCGCCTGAGAGGGAGCTCCGCCCCTGGGGCAGTCTCT

GCCCTCCAGTGCCGGCTGCTCTTCCCGAGAATGGAGTGGGCGCCCGGGGAAGAGAAGGCC

TGGGAGTCGCAGGCGGTGGGGTGCGAAGCCGTGCGAGTGCGAGACCCCAAGACAGCGCCG

AGTCGAGCGCGCCCTCACACTAGGGAACCCGTCTTTGGCCCCAGAAACGAGCCCCTAGCC

CCTCACTGAAGAGCCCACCCCAATTCCCTACGGAAAAGTCCCCCTAGTTCCCCACTTGGG

AACCCACCCGAGACCCTCCTTCCTGGAGGTATTTCCTTTAAGCAAACTACAGCTTTTCCT

CTGCAATCACTGGCTCTGCAGAAAGTCAGA
