# Supplementary material for: An expanded biomarker panel for the detection of prostate cancer from urine DNA
Source: Exp Hematol Oncol. 2019 Jun 27;8:13. doi: 10.1186/s40164-019-0137-x (PMC6598372; doi:10.1186/s40164-019-0137-x)
Supplement: Supplementary file 2 — Additional file 2. The range of average methylation values and the number of methylated markers obtained from DRE and FV DNAs by grade. [file 40164_2019_137_MOESM2_ESM.docx]

**Supplemental Table 3:** The minimum, median, mean, maximum, first and third quartile for the average methylation and the number of methylated markers obtained from DRE and FV DNAs by grade.

| Sample.Type | Biopsy.Result | Measure | N | Min | X1stQu | Median | Mean | X3rdQu | Max |
| --- | --- | --- | --- | --- | --- | --- | --- | --- | --- |
| DRE | 0 | Average Methylation | 49 | 0 | 0.26 | 0.97 | 1.18 | 1.81 | 4.16 |
| DRE | 1+2 | Average Methylation | 33 | 0.35 | 2.4 | 3.17 | 3.77 | 4.67 | 10.17 |
| DRE | 1 | Average Methylation | 15 | 0.35 | 1.43 | 2.51 | 2.37 | 3.07 | 4.12 |
| DRE | 2 | Average Methylation | 18 | 1.34 | 3.13 | 4.32 | 4.94 | 6.69 | 10.17 |
|  |  |  |  |  |  |  |  |  |  |
| DRE | 0 | # of Positive Markers | 49 | 0 | 2 | 5 | 6.31 | 9 | 17 |
| DRE | 1+2 | # of Positive Markers | 33 | 3 | 12 | 16 | 16.36 | 20 | 31 |
| DRE | 1 | # of Positive Markers | 15 | 3 | 7.5 | 13 | 12.13 | 16.5 | 20 |
| DRE | 2 | # of Positive Markers | 18 | 8 | 16 | 17 | 19.89 | 26 | 31 |
|  |  |  |  |  |  |  |  |  |  |
| FV | 0 | Average Methylation | 35 | 0 | 0.51 | 1.06 | 1.25 | 1.83 | 3.06 |
| FV | 1+2 | Average Methylation | 28 | 0.7 | 2.52 | 3.34 | 3.81 | 4.59 | 11 |
| FV | 1 | Average Methylation | 10 | 1.65 | 2.70 | 3.01 | 3.21 | 3.46 | 5.53 |
| FV | 2 | Average Methylation | 18 | 0.7 | 2.18 | 3.90 | 4.14 | 4.78 | 11 |
|  |  |  |  |  |  |  |  |  |  |
| FV | 0 | # of Positive Markers | 35 | 0 | 3.5 | 6 | 6.63 | 9.00 | 18 |
| FV | 1+2 | # of Positive Markers | 28 | 6 | 14.00 | 16 | 17.04 | 20.25 | 31 |
| FV | 1 | # of Positive Markers | 10 | 11 | 14.00 | 15 | 16.40 | 19.00 | 23 |
| FV | 2 | # of Positive Markers | 18 | 6 | 12.00 | 17 | 17.39 | 21.00 | 31 |
